# Effects of early high-dose vasopressor administration in patients after aneurysmal subarachnoid hemorrhage: a retrospective single-center study

**DOI:** 10.1007/s00701-025-06435-5

**Published:** 2025-03-17

**Authors:** Jan Küchler, Niclas Hinselmann, Maria V. Matone, Anastassia Löser, Volker M. Tronnier, Claudia Ditz

**Affiliations:** 1 Department of Neurosurgery, University Hospital of Schleswig-Holstein - Campus Lübeck, Ratzeburger Allee 160, 23538 Lübeck, Germany; 2 Department of Radiation Oncology, University Hospital of Schleswig-Holstein - Campus Lübeck, Ratzeburger Allee 160, 23538 Lübeck, Germany

**Keywords:** Aneurysmal subarachnoid hemorrhage, Cerebral vasospasm, Delayed cerebral ischemia, Norepinephrine, Vasopressin, Vasopressors

## Abstract

**Background:**

Although the use of vasopressors is recommended after aneurysmal subarachnoid hemorrhage (aSAH) to maintain adequate cerebral perfusion pressure, data on potential adverse effects on delayed cerebral ischemia (DCI) are lacking. The aim of this study was to evaluate the effects of early high-dose vasopressor therapy with norepinephrine alone or additional vasopressin on the subsequent occurrence of DCI, DCI-related infarction and functional outcomes.

**Methods:**

Retrospective evaluation of aSAH patients admitted between January 2010 and December 2022. Demographic, clinical and outcome data as well as daily norepinephrine equivalent (NEE) scores were collected. Potential risk factors for DCI, DCI-related infarction and functional outcome 3 months after discharge were assessed by logistic regression analyses.

**Results:**

A total of 288 patients were included. 208 patients (72%) received vasopressor therapy during the first 14 postictal days with a mean NEE score of 3.8 µg/kgBW/h. The highest NEE scores were observed in the acute phase after hemorrhage and mainly in poor-grade patients. The mean NEE score during the postictal days 1–4 was significantly higher in patients who developed DCI or DCI-related infarction and who had an unfavorable functional outcome. Multivariable logistic regression analysis identified a high NEE score on postictal days 1–4 as an independent predictor of DCI and unfavorable functional outcome.

**Conclusions:**

Vasopressor use is common in aSAH patients in the acute phase after hemorrhage. Our results suggest that high NEE scores during the first 4 days after ictus represent an independent prognostic factor and might aggravate the complex cerebral sequelae associated with the disease.

## Introduction

Aneurysmal subarachnoid hemorrhage (aSAH) is a severe subtype of stoke, associated with high morbidity and mortality [35, 48]. Prehospital mortality rates up to 26% have been reported, and approximately 13 to 20% of patients die in the acute hospital stay [14]. About half of survivors are left with some degree of persistent neurological or cognitive deficit [6, 11]. Patients outcome following aSAH is not only attributed to the severity of the initial hemorrhage but also to secondary disease-related complications such as cerebral vasospasm (CVS), delayed cerebral ischemia (DCI), or cardiac injury [12, 27, 38, 51]. These complications may cause disturbances in cerebral blood flow (CBF) leading to a reduction of brain tissue perfusion and oxygenation [36]. Recent guidelines recommend the use of vasoactive drugs to maintain a cerebral perfusion pressure (CPP) sufficient to supply the brain, especially in the presence of CVS and DCI [14].

However, especially in severely affected patients under deep sedation, high vasopressor doses might be required to maintain the target arterial blood pressure (BP). In addition, aSAH is associated with significant systemic complications such as myocardial dysfunction and neurogenic pulmonal edema. The mechanisms related to these complications include increased activation of the sympathetic nervous system with subsequent elevation in levels of circulating catecholamines. Reversible cardiac dysfunction, which is frequently observed at an early stage after acute aSAH [21, 30, 45], is directly related to the severity of the primary brain injury and may aggravate hemodynamic instability [40]. This may further increase the need for high vasopressor doses especially in the early phase after aSAH.

Although the effect of vasopressors on improving mean arterial pressure (MAP) and thereby CPP is well established [37], it is also well known that high-dose vasopressor administration can induce serious end organ damage such as limb or bowel ischemia and acute kidney failure due to the extensive constriction of small peripheral blood vessels [15, 25]. In addition, animal studies suggest that vasoactive drugs might have negative effects on cerebral microvasculature [23, 24, 26]. Thus, high-dose vasopressor use might aggravate cerebral microcirculatory dysfunction after aSAH.

Studies investigating a potential relationship between high-dose vasopressor therapy and the development of DCI, secondary cerebral infarction or clinical outcome are scarce. A recent retrospective study by *Cattaneo et al.* indicates a significant association between higher dose norepinephrine administration and the occurrence of DCI-related infarction after aSAH [5]. The authors found significantly higher cumulative norepinephrine doses during the first 14 days of treatment in those patients who developed DCI-related infarction, and confirmed an independent statistical association after adjusting for potential confounders.

However, during the intensive care treatment of aSAH patients vasopressor dosing might be influenced by several factors such as intravenous or volatile anesthesia or ICU-acquired infections and sepsis, which often only occur in the later phase of the hospital stay and might also have a negative impact on DCI and the patients’ outcome [13, 46]. In addition, vasopressors are often administered to increase CPP when DCI or CVS are clinically suspected. This treatment strategy of ‘induced hypertension (IH)’ represents a relevant potential bias, as high vasopressor doses may only reflect a reaction to a DCI event and not its cause.

To date there are no data on the time course of vasopressor therapy or high-risk periods for vasopressor use after aSAH. In clinical perception, there appears to be a high demand for vasopressors, particularly during the acute and subacute phase following aSAH and especially in severely affected high grade patients (World Federation of Neurosurgical Societies (WFNS) grade 4–5).

With this study, we aimed to investigate the time course of (high) vasopressor therapy during the first 14 days after hemorrhage, and to additionally analyze whether the cumulative vasopressor dose during the subacute phase (days 1–4) after aneurysm rupture represents a prognostic factor for the incidence of DCI-related infarction or unfavorable functional outcome. We have limited our analysis to the early phase after aSAH in order to exclude possible confounding factors for high vasopressor demand, such as IH in response to DCI or symptomatic CVS.

## Patients and methods

### Patient cohort and study design

The medical records of all consecutive aSAH patients who were admitted to our tertiary care university hospital between January 2010 and December 2022 were retrospectively reviewed and analyzed. The diagnosis of acute SAH from a ruptured intracranial aneurysm was confirmed by computed tomography (CT) or lumbar puncture and the presence of an aneurysm in CT angiography and/or digital subtraction angiography (DSA). The study was conducted in accordance with the ethical standards of the Declaration of Helsinki and approved by the ethics committee of the University of Lübeck (reference 2023 − 557). This is an ethical committee-approved retrospective chart review study; all patient information was de-identified, and patient consent to participate or publish is not applicable.

We included patients aged 18 or older who were treated for at least 14 days in our neurointensive care unit. Patients with aSAH caused by an aneurysm associated with arteriovenous malformation (AVM) or mycotic pseudoaneurysms were excluded. Additionally, patients who died during the first 14 days after ictus or who received palliative care only due to poor prognosis were excluded from this analysis. According to the outcome variables analyzed in this study, we defined additional exclusion criteria as follows: (1) delayed presentation with admission more than 3 days after the anamnestic bleeding event, (2) detection of angiographic CVS in the initial DSA indicating previous bleeding, (3) missing data/ incomplete records on vasopressor therapy.

### Data acquisition

Demographic and clinical characteristics of the study cohort as well radiological and outcome data were collected by chart review. Hourly administration of norepinephrine and vasopressin administration was recorded in the first 14 days after aSAH. In particular, vasopressor administration during aneurysm repair in case of bleeding or anesthesiologic complications or during endovascular rescue therapies for CVS due to hypotensive side effects of intraarterial nimodipine therapy was not recorded. Since different vasopressors have different pharmacological characteristics and effects on hemodynamics, we used an updated norepinephrine equivalent (NEE) score proposed by *Kotani et al.* to quantify the vasopressor exposure in our study [22]. The NEE score converts the dose of each vasopressor equivalent to that of norepinephrine (µg/kgBW/h). For vasopressin (U/min), a conversion ratio of 2.5 was used.

### Management of aSAH patients

After aneurysm repair with either surgical clipping or endovascular obliteration within 48 h after admission, patients were monitored in our neurointensive care unit for at least 14 days per institutional aSAH protocol. Vital signs were assessed continuously via bedside monitoring. Follow-up included an early CT scan after intervention (within 24 h after aneurysm treatment) in all patients. Depending on the clinical condition, further follow-up CT and/or perfusion CT (PCT) scans were performed during the hospital stay. In case of acute symptomatic hydrocephalus, cerebrospinal fluid (CSF) drain was placed. Patients received standard medical treatment according to current international guidelines with euvolemia and a target hemoglobin value > 10 g/dl [14].

All patients were treated with nimodipine orally at a standard dosing regimen of 60 mg every 4 h or intravenously with 2 mg of nimodipine per hour. In case of systemic hypotension following oral application, a modified dosing regimen with lower doses at a higher frequency (30 mg nimodipine every two hours) was used [9, 28].

Routine monitoring for DCI and CVS included serial neurologic examinations and daily transcranial Doppler (TCD) sonography. CVS on TCD was defined as mean flow velocity (FV) > 140 cm/s in the anterior circulation, or an increase of FV by ≥ 50 cm/s over 24 h. If patients were clinically assessable, DCI was defined by the clinical criteria set by *Vergouwen et al.* (new focal neurologic deficit or a decrease in the Glasgow coma scale ≥ 2 for at least 1 h, not ascribable to other diagnoses) [50]. In analgosedated patients, further DCI surveillance was performed by placement of invasive neuromonitoring with continuous intracranial pressure (ICP) and brain tissue oxygen monitoring (PBrO_2_, Neurovent-TO^®^ or Neurovent. PTO^®^, Raumedic AG, Helmbrechts, Germany), and repeated PCT imaging. In these patients, the clinical definition of DCI was extended to include events of functional deterioration (functional DCI) as described previously [43, 49]. Functional DCI was defined as cerebral oxygenation crises with refractory decrease in PBrO_2_ below 15 mmHg and/or as DCI-related hypoperfusion on PCT defined as areas of prolonged time to drain (TTD) not related to other causes [10, 52]. If DCI or CVS was suspected, arterial hypertension was induced (induced hypertension, IH) by means of fluid and vasopressor administration. If the medical treatment failed to improve the patients’ condition, DSA was performed to confirm angiographic CVS and to initiate vasospasmolysis, either by intraarterial nimodipine infusion and/or transluminal balloon angioplasty. Angiographic CVS was defined as the occurrence of new arterial vessel narrowing on follow-up DSA not attributable to atherosclerosis, catheter-induced spasm, or vessel hypoplasia.

### Blood pressure management and vasopressors

In the acute postictal phase, in ventilated patients and during vasopressor infusion, arterial BP was continuously monitored invasively via arterial catheter. In clinically stable patients, BP was subsequently monitored with a non-invasive blood pressure cuff. According to our institutional protocol, the initial target systolic BP before securing the aneurysm was < 140 mmHg. After aneurysm repair, the target for MAP was approximately 80–90 mmHg. In case of DCI or CVS, IH was performed with a systolic BP goal of 160–180 mmHg. BP values below the targeted range were first treated with fluid optimization, and then supported with continuous intravenous vasopressor infusion. The vasopressor of choice for BP support was norepinephrine (Noradrenalin Aguettant, 0.08 mg/ml, Laboratoire Aguettant, Lyon, France), administered as continuous intravenous infusion through a central access line. Additive to norepinephrine, vasopressin (Empressin ^®^ 40 I.E./2 ml, AOP Orphan Pharmaceuticals GmbH, Vienna, Austria) was used as needed when target BP values were not achieved.

### Definition of outcome variables

The primary outcome was defined as the patients’ functional outcome assessed using the modified Rankin Scale (mRS 0–6) at 3 months after the initial hemorrhage. For analysis, mRS was categorized into favorable outcome (mRS 0–3) and unfavorable outcome (mRS 4–6). The overall occurrence of DCI-related cerebral infarction was used as a secondary outcome parameter. DCI-related infarction was defined as new hypodense lesions on follow-up CT not attributable to other causes. Pre-existing infarctions and infarctions related to the primary brain damage, cerebral herniation, the initial aneurysm treatment or other iatrogenic interventions were excluded.

### Statistical analysis

Data analysis was performed using the software IBM^®^ SPSS^®^ 28 (IBM; Armonk, NY, USA). Continuous data are presented as medians with interquartile range (IQR), while categorical data are presented by counts and percentages.

Categorical variables were evaluated with the chi-square test or Fisher’s exact test, if applicable. Continuous variables were analyzed using the Mann–Whitney U test for non-normally distributed data. A p value < 0.05 was considered as statistically significant.

To identify independent predictors for different outcome variables, we performed univariable analyses with chi-square testing. To exclude confounding factors, we used a multivariable logistic regression model with stepwise backward selection. Multivariable regression results are reported as odds ratio (OR), 95% confidence interval (CI-0.95), and p-value. Box-Whisker-Plots show median (band), first and third quartiles (box) and 1.5-fold IQR (whisker).

Receiver operating characteristic (ROC) curve analysis was performed and Youden’s index was used to calculate the optimal cut-off value of the mean NEE score (µg/kgBW/h) during postictal days 1–4 for the occurrence of an unfavorable outcome (mRS 4–6) after 3 months.

## Results

### Study population and patient characteristics

We identified 429 aSAH patients admitted to our department between 2010 and 2022. We excluded 6 patients with AVM-associated or mycotic aneurysms. We also excluded 83 patients who died within 14 days after hemorrhage or in whom treatment was withdrawn due to neurological devastation. 33 patients were excluded because of delayed hospital admission more than 3 days after onset of symptoms or because of the detection of angiographic CVS in the initial DSA, indicating a previous hemorrhage. 19 patients were excluded due to incomplete records or missing data.

Ultimately, a total of 288 patients were included in this retrospective analysis. Baseline characteristics and outcome measures of the study cohort are summarized in Table 1. The median patient age was 55 years (IQR 16.3) and the majority of patients were female (*n* = 204, 71%). Ten patients (3%) had diabetes and 109 patients (38%) had a history of arterial hypertension. In our cohort, 180 patients (63%) presented with mild to moderate aSAH (WFNS grade 1–3), while 108 patients (37%) were admitted with severe aSAH (WFNS grade 4–5). The majority of patients (*n* = 264, 92%) had Fisher grade 3–4 blood distribution on the initial cranial CT scan. Decompressive craniectomy was performed in 61 (21%) patients, and 67 patients (23%) required CSF drainage due to acute hydrocephalus. Most ruptured aneurysms were located in the anterior circulation (*n* = 242, 84%) with the anterior communicating artery (*n* = 79, 27%) and the middle cerebral artery (*n* = 75, 26%) as the most common locations. Aneurysms were secured by endovascular treatment in about two thirds of patients (*n* = 197, 68%), and by surgical clipping in 91 patients (32%). Overall DCI-events were observed in 116 patients (40%), and angiographic CVS was diagnosed in 113 patients (39%). DCI-related infarction occurred in 19% of patients (*n* = 54). Twelve patients (4%) of our cohort died during acute hospitalization (after postictal day 14). Favorable functional outcome (mRS 0–3) at 3 months follow-up was achieved in 176 patients (61%).

**Table 1:** Characteristics of the study cohort

Variable	All (*n* = 288)
Age in years, median (IQR)	55 (16.3)
Female sex, n (%)	204 (71)
Diabetes, n (%)	10 (3)
History of hypertension, n (%)	109 (38)
WFNS grade, n (%) 1 2 3 4 5	86 (30) 55 (19) 39 (13) 48 (17) 60 (20)
Fisher scale, n (%) 1 2 3 4	3 (1) 21 (7) 101 (35) 163 (57)
Decompressive craniectomy, n (%)	61 (21)
Intraparenchymal hemorrhage, n (%)	94 (33)
Intraventricular hemorrhage, n (%)	198 (69)
Acute hydrocephalus requiring CSF drain, n (%)	67 (23)
Aneurysm localization, n (%) ICA MCA ACOM ACA PCOM Posterior circulation	47 (16) 75 (26) 79 (27) 23 (8) 18 (6) 46 (16)
Aneurysm obliteration, n (%) Surgical clipping Endovascular treatment	91 (32) 197 (68)
Overall occurrence of DCI, n (%) DIND PBrO_2_ < 15 mmHg PCT hypoperfusion	116 (40) 20 (7) 37 (13) 83 (29)
Angiographic CVS, n (%)	113 (39)
DCI-related cerebral infarction, n (%)	54 (19)
In-hospital mortality, n (%)	12 (4)
Functional outcome after 3 months, n (%) Favorable outcome (mRS 0–3) Unfavorable outcome (mRS 4–6)	176 (61) 112 (29)

*IQR* interquartile range; *WFNS* World Federation of Neurosurgical Societies; *CSF* cerebrospinal fluid;

*ICA* internal carotid artery; *MCA* middle cerebral artery; *ACOM* anterior communicating artery;

*ACA* anterior cerebral artery; *PCOM* posterior communicating artery; *DCI* delayed cerebral ischemia;

DIND delayed ischemic neurological deficit; PBrO_2_ Brain issue oxygen; PCT perfusion computed tomography;

CVS cerebral vasospasm; *mRS* modified Rankin Scale

### Vasopressor treatment

In our study cohort, 208 patients (72%) received vasopressor therapy during the first 14 days of treatment. Of these, all patients received norepinephrine infusion (100%), and 66 patients (23%) received additional vasopressin.

In 54 patients (19%) vasopressin was administer during postictal days 1–4.

During days 1–14 after ictus, the overall median of vasopressor therapy was 9 days (IQR 2–13). The mean NEE score was 3.8 µg/kgBW/h (SD 4.0) during the entire observation period (days 1–14) and 4.9 µg/kgBW/h (SD 5.2) during postictal days 1 to 4.

**Fig. 1:**
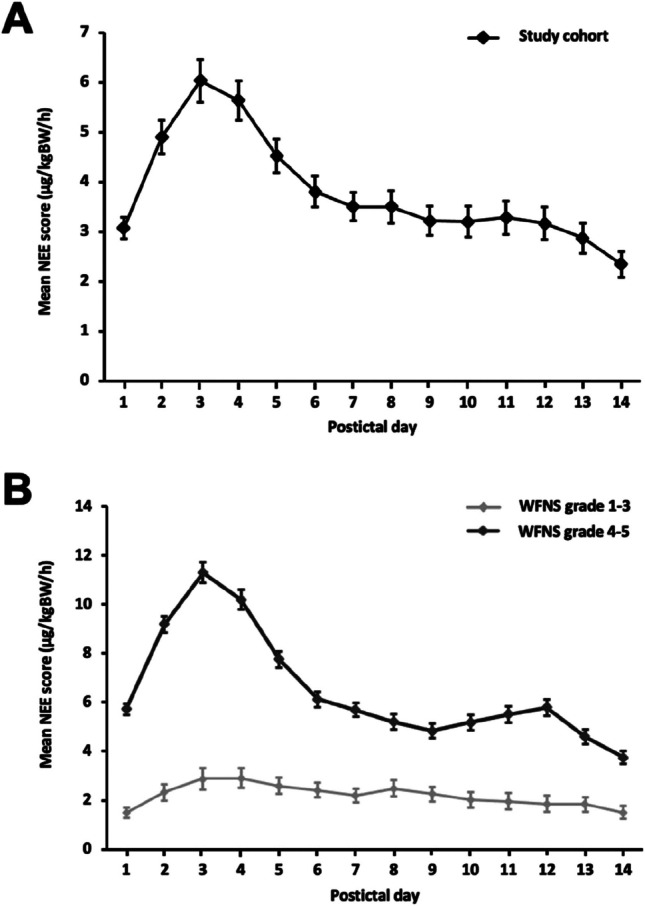
Time course of the daily cumulative NEE score during the first 14 days after hemorrhage in the total study cohort (A) as well as in WFNS grade 1–3 and WFNS grade 4–5 patients. Data are presented as mean with standard error

The time course of vasopressor administration in our cohort during the first 14 days after the bleeding event is shown in Fig. 1. We found a significant steep-wise increase in the NEE score on postictal day 1 with peek-vasopressor doses on postictal day 3. Thereafter, the NEE score values decrease again significantly every day and reach a stable level on the 7th postictal day. The subgroup analysis of patients with WFNS grade 1–3 and patients with WFNS grade 4–5 shows that WFNS grade 4–5 patients are significantly more frequently affected by high-dose vasopressor therapy (Fig. 1, B).

### Effects of high NEE score during postictal days 1–4

**Fig. 2:**
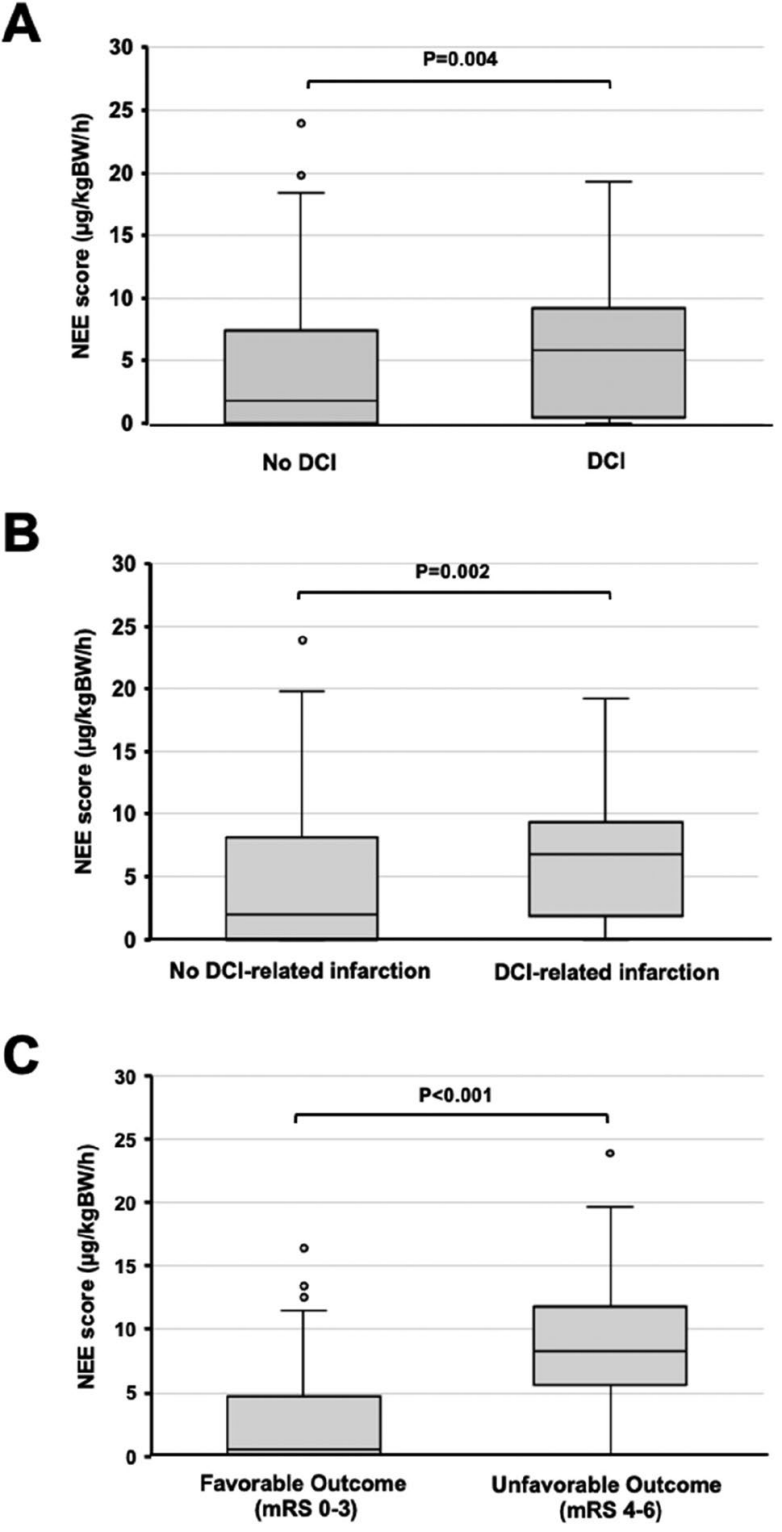
Box plots comparing the mean cumulative NEE score between days 1 to 4 after hemorrhage in patients with and without occurrence of DCI (**A**), DCI-related infarction (**B**) and favorable or unfavorable functional outcome (mRS 0–3 vs. mRS 4–6) at 3 months (**C**)

Mean cumulative NEE score during the postictal days 1–4 was significantly higher in patients who developed overall DCI-events (p=0.004, Fig. 2A), DCI-related infarction (p=0.002, Fig. 2B) and who showed an unfavorable functional outcome (mRS 4–6) at 3 months (p < 0.001, Fig. 2C).

### Risk factors associated with the occurrence of overall DCI

The results of the logistic regression analysis of potential predictors of overall DCI (clinical and functional DCI) are given in Table 2. In our cohort, high NEE score on postictal days 1–4 (OR 1.06, *p* = 0.013) was the only variable that showed a significant association with the occurrence of overall DCI-events.

**Table 2:** Univariable logistic regression analysis of possible clinical predictors of overall DCI

Parameter	Clinical predictors of DCI	
	OR [CI-0.95]	*P* value
Age (years)	0.98 [0.96–1.00]	0.084
Female sex	1.31 [0.78–2.22]	0.312
WFNS grade 4–5	1.57 [0.98–2.53]	0.062
Fisher scale 3–4	2.33 [0.63–8.64]	0.208
Acute hydrocephalus	1.43 [0.85–2.40]	0.174
Aneurysm localization Anterior circulation	0.68 [0.35–1.32]	0.249
Aneurysm treatment Surgical treatment	0.89 [0.54–1.49]	0.669
NEE score postictal days 1–4 (µg/kgBW/h)	**1.06 [1.01–1.11]**	**0.013**

Data comparisons were made using log rank test for univariable analysis

Statistically significant differences are made bold (*p* < 0.05)

*DCI* delayed cerebral ischemia; *OR* odds ratio; *CI* confidence interval; *WFNS* World Federation of Neurosurgical Societies;

*NEE* norepinephrine equivalent

### Risk factors associated with DCI-related infarction

Univariable risk factors for the occurrence of DCI-related cerebral infarction were WFNS grade 4–5 (OR 2.52, *p* = 0.001), acute hydrocephalus (OR 2.76, *p* = 0.002) and a high NEE score on postictal days 1–4 (OR 1.08, *p* = 0.005). After adjusting for confounding variable interaction in a multivariable logistic regression model, only WFNS grade 4–5 (OR 2.07, *p* = 0.021) and acute hydrocephalus (OR 2.61, *p* = 0.021) were confirmed as independent predictor of DCI-related infarction. The detailed analysis can be found in Table 3.

**Table 3:** Logistic regression analysis of possible clinical predictors of DCI-related infarction

	Clinical predictors of DCI-related infarction			
Parameter	Univariable analysis		Multivariable analysis	
	OR [CI-0.95]	*P* value	OR [CI-0.95]	*P* value
Age (years)	1.02 [1.00–1.05]	0.069	-	-
Female sex	0.92 [0.48–1.77]	0.473	-	-
WFNS grade 4–5	**2.52 [1.41–4.51]**	**0.001**	**2.07 [1.09–3.95]**	**0.021**
Fisher scale 3–4	1.67 [0.36–7.72]	0.513	-	-
Acute hydrocephalus	**2.76 [1.37–5.58]**	**0.002**	**2.61 [1.16–5.90]**	**0.021**
Aneurysm localization Anterior circulation	0. 61 [0.24–1.51]	0.193	-	-
Aneurysm treatment Surgical treatment	0.56 [ 0.28–1.12]	0.067		
NEE score postictal days 1–4 (µg/kgBW/h)	**1.08 [1.02–1.13]**	**0.005**	1.04 [0.97–1.12]	0.237

Data comparisons were made using univariable and multivariable logistic regression. Statistically significant differences are made bold (*p* < 0.05)

*DCI* delayed cerebral ischemia; *OR* Odds ratio; *CI* confidence interval; *WFNS* World Federation of Neurosurgical Societies;

*NEE* norepinephrine equivalent

### Risk factors associated with unfavorable functional outcome

The results of the logistic regression model with functional outcome at 3 months as the dependent variable are summarized in Table 4. We found that older age (OR 1.06, *p* < 0.001), WFNS grade 4–5 (OR 8.10, *p* < 0.001), Fisher grade 3–4 (OR 10.50, *p* = 0.003), acute hydrocephalus (OR 2.43, *p* < 0.001) and a high NEE score on postictal days 1–4 (OR 1.32, *p* < 0.001) were significantly associated with unfavorable functional outcome in univariable analysis. Multivariable analysis confirmed older age (OR 1.08, *p* < 0.001), WFNS grade 4–5 (OR 2.23, *p* = 0.029) and high NEE score on postictal days 1–4 (OR 1.29, *p* < 0.001) as independent risk factors for unfavorable functional outcome.

**Table 4:** Logistic regression analysis of possible clinical predictors of unfavorable outcome

	Clinical predictors of unfavorable outcome (mRS 4–6)			
Parameter	Univariable analysis		Multivariable analysis	
	OR [CI-0.95]	*P* value	OR [CI-0.95]	*P* value
Age (years)	**1.06 [1.04–1.09]**	**< 0.001**	**1.08 [1.04–1.11]**	**< 0.001**
Female sex	1.15 [0.69–1.94]	0.340	-	-
WFNS grade 4–5	**8.10 [4.64–14.13]**	**< 0.001**	**2.23 [1.08–4.59]**	**0.029**
Fisher scale 3–4	**10.50 [1.37–80.64]**	**0.003**	6.01 [0.40–92.97]	0.193
Acute hydrocephalus	**2.43 [1.40–4.21]**	**< 0.001**	1.25 [0.61–2.55]	0.549
Aneurysm localization Posterior circulation	0.89 [0.47–1.71]	0.431	-	-
Aneurysm treatment Surgical treatment	1.61 [0.98–2.70]	0.069	-	-
NEE score postictal days 1–4 (µg/kgBW/h)	**1.32 [1.24–1.42]**	**< 0.001**	1.29 [1.18–1.40]	**< 0.001**

Data comparisons were made using univariable and multivariable logistic regression. Statistically significant differences are made bold (*p* < 0.05)

*mRS* modified Rankin Scale; *OR* Odds ratio; *CI* confidence interval; *WFNS* World Federation of Neurosurgical Societies;

*DCI* delayed cerebral ischemia; *NEE* norepinephrine equivalent

**Fig. 3:**
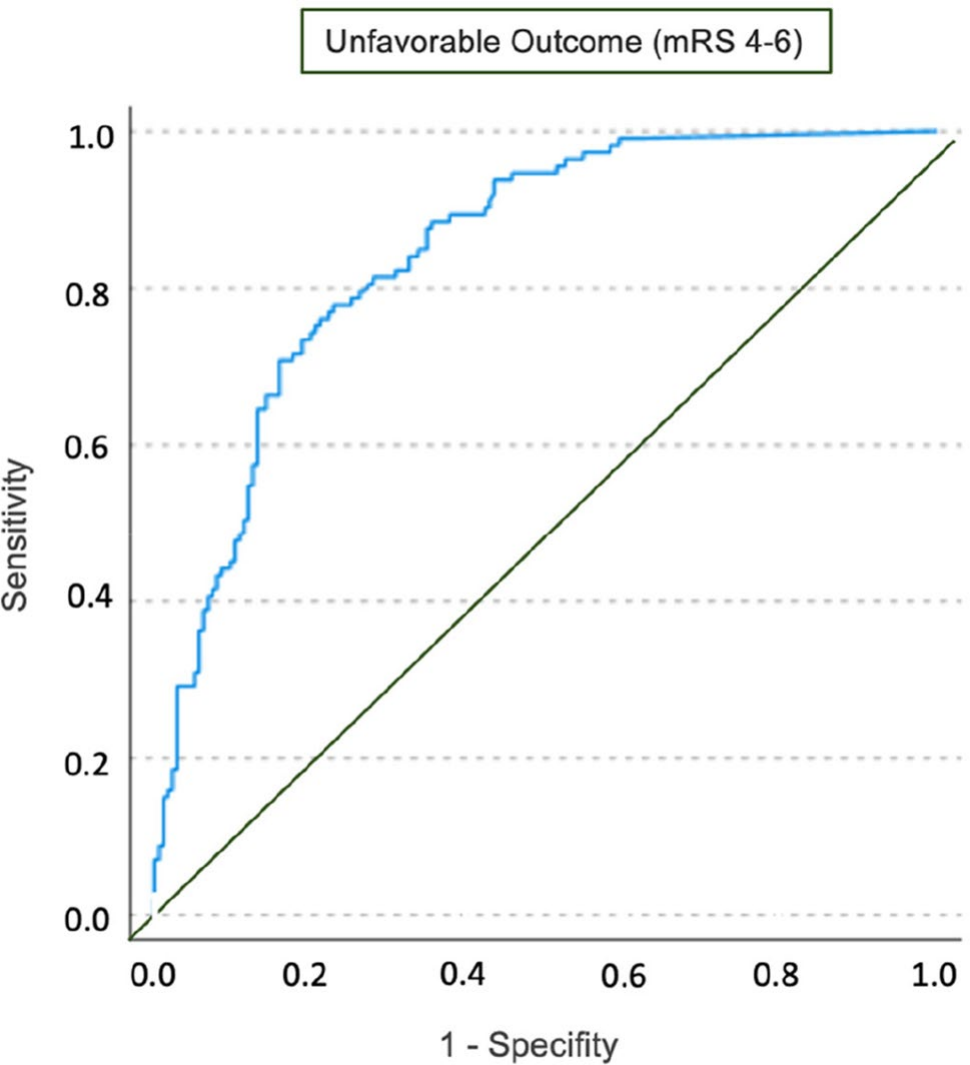
Receiver operating characteristic **(**ROC) analysis of the association between the mean cumulative NEE score between days 1 to 4 after hemorrhage and the occurrence of an unfavorable outcome (mRS 4–6)

ROC analysis showed that the mean NEE score during postictal days 1–4 differed significantly between patients with favorable and unfavorable outcomes (AUC = 0.846, 95% CI 0.802–0.890, *p* < 0.001, Fig. 3). Patients who received a NEE score of more than 5.16 µg/kgBW/h were more likely to have an unfavorable outcome (Youden’s index = 0.55, sensitivity 0.78, specificity 0.77).

## Discussion

We found that hemodynamic augmentation by vasopressors is a common strategy following aSAH and affects nearly three quarters of patients. The results of our study support the clinical observation that high doses of vasopressors are administered especially in the acute and subacute phase after hemorrhage. Hemodynamic instability occurs mainly in the early phase after aSAH and particularly affects patients with high WFNS-grades.

Arterial hypotension during the early phase after aSAH might be influenced by several factors, especially the severity of early brain injury. In addition, aSAH patients are at high risk of cardiac complications including cardiomyopathy, arrhythmia and systolic dysfunction [19]. There are some indications in the literature, that high grade patients are more likely to be affected by severe cardiac dysfunction. The Hunt–Hess grading score shows that electrocardiographic changes are observed consistently at the lower grades, but echocardiography changes such as ventricular wall motion abnormalities and left ventricular diastolic dysfunction are found predominately in patients with higher grades [16, 17, 20]. Moreover, reversible cardiac dysfunction often occurs early after acute aSAH [21, 40, 45] and generally resolves within days to weeks [3]. Patients with high WFNS-grade aSAH and impaired consciousness are additionally more affected by arterial hypotension, as they require deep and prolonged sedation for neuroprotection. Blood loss in the course of surgical aneurysm treatment or decompressive craniectomy might also aggravate hemodynamic instability in severely affected patients. In the early postictal phase, hemodynamic instability can also be aggravated by the start of nimodipine treatment. Recent studies have shown that arterial hypotension after nimodipine administration is generally more common in high grade patients [18, 41]. The further time course of vasopressor therapy after the postictal phase (day 1–4) showed a steady course without a renewed increase in catecholamine requirements. Potential effects of IH during the DCI risk phase or secondary systemic complications such as hospital-acquired infections are not reflected in the dosing course of vasopressor use.

Our results show that a higher cumulative NEE score during the postictal days 1–4 is significantly associated with the occurrence of overall DCI as well with the incidence of secondary DCI-related cerebral infarction. In multivariable analysis, we identified a high NEE score in the early phase after aSAH as an independent risk factor for the occurrence of DCI events. With regards to the incidence of DCI-related cerebral infarction, we also found a significant association in univariable analysis, but this result was not confirmed after adjusting for potential confounders. Studies investigating the relationship between high-dose vasopressor use and the development of DCI after aSAH are scarce. Recently, *Cattaneo et al.* described a significant association between high-dose norepinephrine administration and the occurrence of DCI defined as radiologic evidence of delayed cerebral infarction [5]. In contrast to our study, the authors analyzed the cumulative norepinephrine dose given during the first 14 days of treatment. This time interval includes the high-risk period for the development of DCI and CVS. Thus, the results might be influenced by the implementation of IH as the first-line treatment in case of DCI or CVS. In such cases, high vasopressor doses may only be a reaction to a DCI event and not the cause. However, the results of our study, in which we explicitly examined the postictal phase after aSAH to exclude such bias, confirm an independent association between high-dose vasopressor therapy and the occurrence of clinical and functional DCI events. Since current studies indicate that the pathophysiological mechanisms of early brain injury (EBI) are linked to those of DCI [1, 2], early vasopressor therapy might have a negative impact on the multifactorial cerebral mechanisms of DCI. However, we could not demonstrate an independent correlation between high vasopressor doses in the early postictal phase and the incidence of DCI-related cerebral infarction. Our results suggest that the pathophysiological cascades of DCI development might be triggered by high vasopressor doses in the early phase of the disease, but this does not appear to lead to an increased risk of delayed secondary infarction.

Recent guidelines do not provide specific recommendations on which vasopressor should be utilized in patients with acute brain injury. Currently, the most commonly used vasopressors are norepinephrine, phenylephrine, and dopamine [37, 53]. In clinical routine, a second vasopressor is often added if blood pressure targets are not achieved with one agent. At our institution, we exclusively use norepinephrine as the first line vasopressor after aSAH. The second-line agent is usually vasopressin which is used in addition to norepinephrine as a catecholamine-sparing agent to reduce the levels of norepinephrine dosage [34]. In our cohort, only 23% of patients were treated with vasopressin as a second vasopressor. The majority of patients received norepinephrine as the only vasoactive agent for MAP maintenance. Studies indicating a relationship between high-dose norepinephrine and the development of DCI are still limited. A direct link of DCI due to the administration of noradrenaline has only been demonstrated in a case report to date [55].

However, experimental animal studies suggest that norepinephrine causes direct vasoconstriction in cerebral microvessels with a consecutive decrease in oxygen saturation in the cerebral microvasculature [24, 31]. In addition, several clinical studies have found a correlation between the use of vasopressors and a decrease of cerebral oxygenation, which is attributed to microcirculatory vasoconstriction [4, 7, 42, 54]. High-dose norepinephrine therapy could therefore increase the risk of irreversible infarction, especially in the high-risk period for delayed ischemia between postictal days 4 and 14 and in brain regions that are already hypoperfused due to concomitant DCI and CVS.

In terms of functional outcome, the available literature on the use of various vasoactive agents in patients with aSAH provides controversial results. *Roy et al.* analyzed the initial choice of vasopressors used for IH in patients with aSAH and DCI and compared the effects of phenylephrine and norepinephrine on efficacy, adverse effects, and outcome. The authors found better clinical outcomes in patients started with norepinephrine [39]. These results are in contrast to a more recent nationwide cohort study from the United states on 2634 patients with nontraumatic SAH which evaluated the association between initial vasopressor choice and mortality. In this study, phenylephrine was significantly associated with reduced mortality compared to norepinephrine and dopamine [53].

Our results provide first evidence that high vasopressor dosing in the early phase after aSAH represents a significant risk factor for an unfavorable functional outcome after 3 months. Since patients with high WFNS grades are particularly affected by early high-dose vasopressor therapy, it is reasonable to assume that our result might be influenced by the severity of the initial bleeding event. Since the initial presentation is a strong predictor of functional outcome, high vasopressor demand in the early phase might be prognostically synonymous with a high WFNS grade. However, multivariable analysis confirmed a ‘high NEE score’ as well as ‘older age’ and ‘high WFNS grade’ as independent predictors for unfavorable functional outcome. As our results suggest that a high NEE score early after ictus is not related to an increased incidence of DCI-associated infarction, other pathophysiological aspects must be discussed to explain the correlation between high vasopressor use and poor functional outcome. Previous studies found elevated catecholamine levels in the cerebrospinal fluid of patients with high-grade aSAH and report a correlation with poor clinical outcome [8, 32]. Disturbances of the blood-brain barrier, which is frequently observed after severe aSAH, could favor excessive entry of norepinephrine into the subarachnoid space which could thus contribute to the deleterious effects of endogenous catecholamines, especially in high grade patients.

In addition, aSAH patients are at high-risk of cardiac complications associated with pronounced acute sympathetic activation and consecutive release of several endogenous catecholamines including norepinephrine. The severity of cardiac dysfunction is related to the extent of catecholamine release and the severity of SAH and mainly affects patients with high grade aSAH [33, 40]. Studies report an independent association between cardiac complications and an increased risk of death, DCI and poor outcome after aSAH [17, 47]. Preclinical and clinical evidence suggests that endogenous catecholamines are rapidly released within minutes of hemorrhage and that myocardial damage and catecholamine levels resolve in the first postictal week [29, 40, 44]. High-dose administration of noradrenaline in the acute postictal phase could add to the deleterious effects of the endogenous catecholamine surge and thus adversely affect the patient’s outcome.

A major limitation of this study is its retrospective approach, which is inherently associated with selection bias and potentially inaccurate data collection. The rate of vasopressor infusion was recorded hourly. However, bolus-dose administration as a temporary measure for transient hypotension was not included in this analysis. In the absence of clinical guidelines, the vasopressor choice varies in aSAH patients and depends on institutional preferences, limiting the generalizability of our results. In particular, there is limited evidence regarding the use of vasopressin as a second-line agent for hemodynamic support in aSAH patients.

We did not analyze MAP or CPP values, as these variables were controlled by our institutional protocol, and did not evaluate the impact of potential confounders on hemodynamics, such as volume status, depth of sedation and/or the occurrence of hospital-acquired infectious complications. The prophylactic administration of the calcium antagonist nimodipine can influence the vasopressor dose due to its hypotensive side effects. We did not analyze the effects of nimodipine dose modifications or the effects of endovascular spasmolysis with bolus application of intra-arterial nimodipine on vasopressor requirement. Adverse events and potential end-organ damage such as kidney injury caused by vasopressor therapy were also not recorded. Additionally, we were unable to systematically evaluate clinical signs cardiac injury such as arrhythmia, serum myocardial enzymes, and diastolic and systolic dysfunction. Therefore, it cannot be ruled out that undiagnosed aSAH-associated cardiac injury may have led to an increased vasopressor requirement or influenced the patient’s outcome. However, we found no documentation of serious cardiac events in the included patients.

## Conclusions

Hemodynamic support by the use of vasopressors is common following aSAH and affects mainly patients with high WFNS-grades. High-dose vasopressor administration occurs primarily in the acute postictal phase after hemorrhage. In our cohort, we observed an independent association between high vasopressor doses on postictal days 1 to 4 and the occurrence of DCI-events and an unfavorable functional outcome after 3 months.

Our results suggest that early high-dose vasopressor use represents an independent prognostic factor after aSAH and might aggravate the complex detrimental pathomechanisms associated with the disease. Prospective randomized controlled studies are required to validate these results and to further evaluate the effects of different vasopressors in aSAH patients.

## Data Availability

No datasets were generated or analysed during the current study.

## Abbreviations

aSAH: Aneurysmal subarachnoid hemorrhage
AVM: Arteriovenous malformation
BP: Blood pressure
BW: Body weight
CBF: Cerebral blood flow
CI: Confidence interval
CPP: Cerebral perfusion pressure
CSF: Cerebrospinal fluid
CT: Computed tomography
CVS: Cerebral vasospasm
DCI: Delayed cerebral ischemia
DSA: Digital subtraction angiography
FV: Flow velocity
ICP: Intracranial pressure
IH: Induced hypertension
IQR: Interquartile range
MAP: Mean arterial pressure
mRS: Modified Rankin Scale
NEE: Norepinephrine equivalent
OR: Odds ratio
PBrO_2_: Brain tissue oxygenation
PCT: Perfusion computed tomography
ROC: Receiver operating characteristic
TCD: Transcranial Doppler
TTD: Time to drain
WFNS: World Federation of Neurosurgical Societies
